# Relationship of neonatal hypothermia and hypoglycemia in late preterm and term born neonates

**DOI:** 10.1186/s40348-025-00204-1

**Published:** 2025-10-14

**Authors:** Calvin Kurz, Marcia Roeper, Alena Welters, Ertan Mayatepek, Thomas Meissner, Sebastian Kummer, Henrike Hoermann

**Affiliations:** Department of General Pediatrics, Neonatology and Pediatric Cardiology, Medical Faculty, University Hospital Düsseldorf, Heinrich-Heine-University, Düsseldorf, Germany

**Keywords:** Neonatal hypothermia, Neonatal hypoglycemia, Late preterm infants, Term neonates, Glucose homeostasis, Thermal regulation

## Abstract

Neonatal hypothermia and hypoglycemia are among the most common clinical disturbances in the immediate postnatal period and contribute to short- and long-term morbidity. While both conditions have been well described individually, particularly in preterm infants, their interaction remains poorly understood and is often underrecognized in clinical practice. In this review, we describe potential pathomechanisms and clinical evidence linking hypothermia and hypoglycemia. We review established risk factors/vulnerable populations for both, i.e. prematurity, being born small for gestational age, or insufficient postnatal energy intake. We discuss potential interventions and susceptible windows for thermal management during postnatal adaption and align these with existing guidelines. Although most available studies are observational in nature and do not allow robust conclusions about causality, they support a plausible and clinically relevant association between hypothermia and hypoglycemia. Therefore, further research, particularly prospective clinical studies comparing actual standard of care with more advanced concepts of thermal management might provide evidence-based guidance for clinical practice and improve neonatal outcomes.

## Introduction

Neonatal hypothermia and neonatal hypoglycemia are two of the most common disorders in the early postnatal period. As both conditions can result in severe metabolic and neurological complications, they represent a significant challenge for neonatal care worldwide [1, 2].

The World Health Organization (WHO) defines hypothermia as a core body temperature < 36.5°C and classifies it into mild (36.0–36.4°C), moderate (32.0–35.9°C) and severe hypothermia (< 32.0°C) [3]. Despite WHO´s recommendations on thermal protection strategies, which have been in place for over two decades, neonatal hypothermia remains a widespread problem [3].

To date, most research has focused on thermoregulation in very preterm infants. Studies have shown that between 44% and 67% of neonates with a birth weight below 1,500 g experience hypothermia (< 36.5°C) upon admission to the neonatal intensive care unit (NICU) [4–6]. In this population, a decrease of 1°C in admission body temperature has been associated with a 28% increased risk of mortality [7, 8]. In contrast, data on the thermoregulation of late preterm and term born neonates are much scarcer, particularly in high-income countries. However, existing data indicate that hypothermia represents a significant problem for late preterm and term neonates. Reported incidence rates of neonatal hypothermia in this population vary substantially, ranging from 21.7% to 83% with markedly higher frequencies observed in low- and middle-income countries [1, 9–11].

An important, yet underrecognized complication of neonatal hypothermia is hypoglycemia. Although a universal blood glucose threshold remains controversial, most clinical guidelines apply operational thresholds between 2.2 and 2.6 mmol/L (40–47 mg/dL), depending on gestational age, postnatal age and clinical presentation [12–14]. Postnatal hypoglycemia affects approximately 15% of all newborns and represents the most common metabolic disturbance in neonates [12]. This is associated with an increased risk of adverse neurodevelopmental outcome [12, 15, 16].

Several observational studies and clinical trials have identified hypothermia as a risk factor for neonatal hypoglycemia [17–22]. However, the causal relationship between these two conditions is not well understood, and their interaction has rarely been investigated in detail. Notably, some studies on neonatal hypothermia do not report concurrent hypoglycemia, and only a subset of national hypoglycemia guidelines acknowledges hypothermia as a relevant risk factor for developing hypoglycemia or indication to screen for hypoglycemia [9, 13]. This lack of consistent evidence and limited clinical awareness is suboptimal, as both conditions frequently present with no overt clinical signs in neonates and may exacerbate one another if not identified and treated promptly [23]. Accordingly, a better understanding of the association between these conditions—particularly in late preterm and term neonates—is of high clinical relevance.

The objective of this review is to synthesize and critically appraise the existing literature regarding the relationship between neonatal hypothermia and hypoglycemia in late preterm and term neonates, to evaluate its implications for clinical management, and identify priorities for future research.

## Methods

We did a comprehensive literature search in PubMed between January 2025 and June 2025, using predefined keywords and combinations thereof, including “neonatal hypothermia”, “neonatal hypoglycemia”, “late preterm”, “term infant”, “hypoglycemia risk factors” and “neonatal thermoregulation”. The search was limited to peer-reviewed articles published in English. The oldest included study was published in 1971, while the majority of the included literature originated from the year 2000 onwards. Titles and abstracts were screened for relevance, and full texts of potentially eligible studies were assessed. In addition, reference lists and internal links within the selected publications were screened for further relevant studies. Because this snowballing approach extended beyond the initial PubMed search, no PRISMA flowchart can be provided.

## Pathophysiological mechanisms linking hypothermia and hypoglycemia

The pathophysiological relationship between hypothermia and hypoglycemia in neonates is multifactorial as well as bidirectional. After birth, infants are exposed to cold stress, as the ambient temperature is significantly lower than in utero [3, 24]. Heat is primarily lost through evaporation of amniotic fluid. Immature skin and a large surface-area-to-body-mass ratio further accelerate this process and increase the risk of hypothermia [25]. Additional heat loss occurs via conduction to cooler surfaces, convection through circulating air, and radiation to colder surrounding objects [3, 18, 24, 25].

In response to hypothermia, neonates activate non-shivering thermogenesis, primarily via brown adipose tissue, which markedly increases metabolic rate and glucose consumption [26]. This heightened energy demand can rapidly deplete hepatic glycogen stores, especially in late preterm and at-risk term infants (e.g., small for gestational age (SGA), infants of diabetic mothers (IDM)) who often have limited glycogen reserves [22, 27–29]. Simultaneously, hypothermia may impair hepatic gluconeogenesis and glycogenolysis by inhibiting key enzymatic processes, further reducing endogenous glucose production [28].

Conversely, hypoglycemia can impair thermogenesis, as glucose is one of the most important substrates for heat production [30]. A lack of sufficient glucose availability can affect the central and peripheral mechanisms regulating body temperature, potentially aggravating hypothermia. These reciprocal effects create a vicious cycle, in which hypothermia and hypoglycemia potentiate one another and increase the risk of clinical deterioration if not promptly identified and treated [1, 22].

## Clinical evidence for the association between hypothermia and hypoglycemia

A growing number of clinical observations have explored the association between neonatal hypothermia and postnatal hypoglycemia. While several studies—predominantly in early preterm neonates—suggest a potential link, evidence remains heterogeneous, and causal mechanisms are not yet fully understood. More recently, attention has expanded to include late preterm and term neonates, but systematic, high-quality data in these groups remain limited. Observational studies from various geographic and socioeconomic contexts support a temporal association between thermal instability and hypoglycemia in the early postnatal period. However, extent and nature of this relationship, as well as its clinical implications, require further clarification. This underscores the relevance of the previously described pathophysiological interplay between thermal dysregulation and impaired glucose homeostasis.

Table 1 provides a structured overview of the 19 most relevant studies between 1993 and 2024 about the co-occurrence and potential association between neonatal hypothermia and hypoglycemia in late preterm and term neonates. These studies vary widely in design, sample size, population, and geographic setting, encompassing both low- and high-income countries. Despite this heterogeneity, a recurring observation emerges: neonates who are hypothermic, especially during the first hours of life, are significantly more likely to develop hypoglycemia.

In a retrospective study conducted in Taiwan, hypoglycemia occurred in 33.3% of at-risk neonates (including SGA, large for gestational age (LGA), and IDM). Importantly, the rate of hypothermia was significantly higher in the hypoglycemic subgroup compared to euglycemic controls (25.7% vs. 16.9%, *p* = 0.029) [21]. Similar results were reported by Nguyen et al. in Canada, where hypothermic neonates exhibited a significantly increased risk of hypoglycemia both in the first 2 h (11.9% vs. 2.8%, *p* < 0.001) and the first 12 h (15.3% vs. 5.9%, *p* = 0.003) of life [18]. In a retrospective analysis from Australia, nearly half (49.5%) of neonates with hypoglycemia were also hypothermic [31]. In Serbia, Lazic-Mitrovic et al. found that 65% of term infants with intrauterine growth restriction (IUGR) developed hypothermia, of whom 53.8% also experienced hypoglycemia compared to 24% in the normothermic control group (*p* < 0.01), underscoring the compounded vulnerability of this subgroup [28]. All these data indicate a frequent co-occurrence of these conditions, although the temporal and causal relationship remains unclear.

Several studies conducted in resource-limited settings further highlight the clinical relevance of this association. In a case-control study from Ethiopia, hypothermic neonates had a 5.5-fold increased likelihood of developing hypoglycemia (84.3% of hypoglycemic neonates were hypothermic vs. 36.7% of euglycemic neonates, adjusted odds ratio (AOR) = 5.485; 95% CI: 2.36–12.75) [17]. Similarly, Sertsu et al. found that the odds of hypoglycemia were nearly three times higher among hypothermic neonates in their retrospective cohort (51.2% vs. 16.4%, AOR = 2.65; 95% CI: 1.22–5.75) [20]. Comparable associations were reported from Ethiopia by Abuhay et al. (38.7% vs. 9.7%, AOR = 2.58; 95% CI: 1.27–5.23) and Fantahun et al. (AOR = 2.06; CI: 1.01–4.26), further supporting the link between thermal instability and impaired glucose regulation [29, 41]. These findings are corroborated by data from Iran, Nepal, China, and India, which collectively suggest that even mild to moderate hypothermia can significantly compromise the neonatal metabolic stability [19, 33–36, 38].

Nevertheless, evidence is not entirely uniform. Some studies, including those by Petersen et al. and Pal et al., did not observe a statistically significant association between the two conditions [32, 39].

These inconsistencies may reflect methodological limitations, such as variability in measurement timing, inconsistent definitions of hypothermia and hypoglycemia, or insufficient sample sizes to detect smaller effect sizes. These limitations make it difficult to compare the majority of existing studies (Table 1).

**Table 1:** Overview of studies examining the relationship between postnatal hypoglycemia and hypothermia

Study author, year	Study region^*^	Study Design	Number of Participants	Study Population	Gestational Age (GA in weeks, mean (SD)) & Birth weight (BW in kg, mean (SD))	Rate of Hypothermia in %	Rate of Hypoglycemia in %	Definition of Hypoglycemia & Hypothermia	Conclusion
Saw et al., 2021 [31]	High-income country, Australia	Retrospective audit	105	Neonates born ≥ 37 weeks of gestation with lowest BG level of less than 2.6 mmol/L	GA: 38 + 2 BW: 3.36	49.5% of all hypoglycemic neonates were hypothermic	12.6% of the screened population had a primary diagnosis of hypoglycemia	Hypoglycemia: < 2.6 mmol/L Hypothermia: < 36.5°C	Hypothermia was present in 49.5% of hypoglycemic neonates; 22.8% had temperatures < 36°C on admission, indicating frequent co-occurrence but unclear causality
Petersen et al., 2024 [32]	High-income country, Germany	Prospective observational cohort study	616	Spontaneous term births with accidental hypothermia	GA: no details (n.d.) BW: n.d.	5.5	12.7	Hypoglycemia: < 2.5 mmol/L Hypothermia: < 36.0°C	Accidental hypothermia was not associated with hypoglycemia in term neonates (0% in hypothermic vs. 15.2% in normothermic group, *p* = 0.072)
Nguyen et al., 2022 [18]	High-income country, Canada	Retrospective chart review	440	Neonates born ≥ 34 + 0 weeks of gestation	GA: 39.3 (1.5) BW: 3.37 (0.53)	26.8	14.7 in the first 2 h 21.2 in the first 12 h	Hypoglycemia: < 2.6 mmol/L Hypothermia: < 36.5°C	Hypothermia within the first 6 h increased the risk of hypoglycemia in the first 2 h (11.9% vs. 2.8%, *p* < 0.001) and 12 h (15.3% vs. 5.9%, *p* = 0.003), and of IV glucose therapy (5.1% vs. 0.9%, *p* = 0.014)
Chen et al., 2022 [21]	High-income country, Taiwan	Retrospective chart review	444	SGA, LGA and IDM late preterm and term neonates	GA: 38.5 (1.24) BW: 2.94 (0.64)	19.8	33.3 (in the at-risk group)	Hypoglycemia: < 2.8 mmol/L Hypothermia: < 36.5°C	Hypothermia occurred in 25.7% of hypoglycemic vs. 16.9% of euglycemic neonates (*p* = 0.029)
Zhao et al., 2020 [19]	Upper-middle-income country, China	Retrospective case-control study	270	Analysis of associated risk factors in 135 neonates with hypoglycemia and 135 healthy neonates	GA: n.d., 15.9% preterm BW: 3.30 (0.55)	12.6	50 (due to study design)	Hypoglycemia: < 2.2 mmol/L Hypothermia: < 36.5°C	70.6% of hypothermic neonates were hypoglycemic vs. 47.0% of normothermic neonates (*p* = 0.010)
Lazic-Mitrovic et al., 2010 [28]	Upper-middle-income country, Serbia	Retrospective observational cohort study	143	Term born neonates with IUGR	GA: 39.02 (1.28) BW: 2.34 (0.3)	65	43.4	Hypoglycemia: < 2.6 mmol/L Hypothermia: < 36.5°C	Among IUGR term neonates, 53.8% of hypothermic infants had hypoglycemia compared to 24% of normothermic infants (*p* < 0.01)
Zayeri et al., 2005 [33]	Upper-middle-income country, Iran	Prospective observational cohort study with longitudinal design	1952	Random selection of 1952 neonates using a multistage sampling technique	GA: n.d., 20.1% preterm BW: n.d., 4.2% VLBW, 26.9% LBW, 68.9% NBW	33.8	19.1	Hypoglycemia: n.d. Hypothermia: < 36.0°C	Hypoglycemia occurred in 12.1% of hypothermic neonates vs. 7.0% of normothermic neonates (AOR = 1.83; 95% CI: 1.33–2.50)
Nayei et al., 2006 [34]	Upper-middle-income country, Iran	Prospective observational cohort study	940	Neonates admitted to the neonatal ward	GA: n.d., 42.8% preterm BW: n.d., 9.7% VLBW, 21.9% LBW, 63.8% NBW	53.3	21.2	Hypoglycemia: < 2.5 mmol/L Hypothermia: < 36.5°C	Hypoglycemia was more frequent in hypothermic neonates (23.7%) than in normothermic neonates (18.4%), without statistical significance (*p* = 0.052)
Sasidharan et al., 2004 [35]	Lower-middle-income country, India	Prospective observational study	604	Every fifth newborn enrolled; severely ill neonates excluded	GA: n.d., 7% preterm BW: n.d., 18.2% SGA	7.62	21.2	Hypoglycemia: < 2.2 mmol/L Hypothermia: n.d.	Hypoglycemia occurred in 60.9% of hypothermic newborns vs. 21.2% of the overall population (*p* = 0.0001)
Bhand et al., 2014 [36]	Lower-middle-income country, India	Prospective observational study	100	Hypoglycemic neonates admitted to the unit	GA: n.d., 49% preterm, 41% term, 9% postterm BW: n.d., 26% LBW	25	Hypoglycemia was a criterion for study inclusion	Hypoglycemia: < 2.5 mmol/L Hypothermia: n.d.	25% of neonates with hypoglycemia were hypothermic
Ochoga et al., 2018 [37]	Lower-middle-income country, Nigeria	Prospective descriptive study	168	Neonates admitted to the special care baby unit within first 24 h post delivery	GA: 37.8 (3.0) BW: 3.2	18.5	11	Hypoglycemia: < 2.2 mmol/L Hypothermia: < 36.5°C	Hypoglycemia occurred in 22.6% of hypothermic neonates compared to 6.3% of normothermic neonates (*p* = 0.010)
Anderson et al., 1993 [38]	Lower-middle-income country, Nepal	Cross-sectional study (CSS, single BG measurement within 50 h postnatal) & longitudinal study (LS, Multiple measurements within 50 h postnatal)	CSS: 226 LS: 31	Uncomplicated term born neonates	CSS: GA: 38.8 (1.6) BW: 2.74 (0.45) LS: GA: 38.8 (1.4) BW: 2.72 (0.39)	CSS: 23.9 LS: 81	CSS: 38 LS: 87	Hypoglycemia: < 2.6 mmol/L Hypothermia: < 35.5°C	57% of hypothermic neonates were hypoglycemic vs. 32% of normothermic neonates (*p* < 0.001)
Pal et al., 2000 [39]	Lower-middle-income country, Nepal	Cross-sectional study (with unmatched case-control analysis)	578	Uncomplicated term born neonates	GA: n.d. BW: 2.69 (0.4)	20	52.6	Hypoglycemia: < 2.6 mmol/L Hypothermia: < 36.0°C	No significant link between hypothermia and hypoglycemia was found; however, hypothermia was associated with reduced ketone availability
Alebachew et al., 2019[40]	Low-income country, Ethiopia	Cross-sectional study	403	Neonates within 6 h of delivery	GA: 38 (2.52) 24,6% preterm BW: 2.94 (0.63)	66.3 (95% CI: 61.1–70.5%)	8.2	Hypoglycemia: n.d. Hypothermia: < 36.5°C	90.4% of neonates with health issues were hypothermic; 31.7% of these conditions were attributed to hypoglycemia
Fantahun et al., 2020 [41]	Low-income country, Ethiopia	Cross-sectional study	196	Neonates admitted to the NICU with age less than 48 h at admission	GA: n.d., 35.7% preterm, 61.7% term, 2.6% postterm BW: n.d., 32.7% LBW, 12.2% VLBW, 52.5% NBW, 2% HBW	43.4	25	Hypoglycemia: < 2.6 mmol/L Hypothermia: < 36.0°C	Neonates with moderate to severe hypothermia were 2.06 times more likely to develop hypoglycemia (AOR = 2.06; 95% CI: 1.01–4.26, *p* < 0.05)
Abuhay et al., 2022 [29]	Low-income country, Ethiopia	Cross-sectional study	497	Neonates admitted to the NICU	GA: n.d., 43.3% preterm, 54.6% term, 2.1% postterm BW: n.d., 44.6% LBW, 53.1% NBW, 2.3% HBW	60.0	27.2	Hypoglycemia: < 2.2 mmol/L Hypothermia: < 36.5°C	Hypoglycemia occurred in 38.7% of hypothermic vs. 9.7% of normothermic neonates (AOR = 2.58; 95% CI: 1.27–5.23)
Sertsu et al., 2022 [20]	Low-income country, Ethiopia	Cross-sectional study	698	Neonates admitted to the NICU	GA: n.d., 32.1% preterm, 67.5% term, 0.4% postterm BW: n.d., 37,8% LBW	36.0	21.2	Hypoglycemia: < 2.2 mmol/L Hypothermia: < 36.5 °C	51.2% of hypothermic neonates developed hypoglycemia compared to 16.4% of normothermic neonates (AOR = 2.65; 95% CI: 1.22–5.75)
Wodajo et al., 2024 [17]	Low-income country, Ethiopia	Unmatched case-control study	249	Neonates admitted to the NICU	GA: n.d., 33.3% preterm BW: n.d., 31.3% LBW	52.6	33.3	Hypoglycemia: < 2.6 mmol/L Hypothermia: < 36.5°C	84.3% of neonates in the hypoglycemic group were hypothermic vs. 36.7% in the euglycemic group, indicating a 5.5-fold increased risk
Kebede et al., 2024 [22]	Low-income country, Ethiopia	Cross-sectional study	400	Neonates admitted to NICU	GA: ∼ 37.0 (3.56), 38.9% preterm BW: n.d., 53.9% LBW, 2.56% VLBW	25	23.5	Hypoglycemia: < 2.6 mmol/l Hypothermia: < 36.5°C	Hypothermic neonates had a 4.4-fold increased risk of prolonged hypoglycemia at 48–72 h (AOR = 4.41; 95% CI: 2.72–10.92).

*Abbreviations*: *AOR* Adjusted odds ratio, *BG* Blood glucose, *BW* Birth weight, *CI* Confidence interval, *CSS* Cross-sectional study, *ELBW* Extremely low birth weight (< 1000 g), *GA* Gestational age, *HBW* High birth weight (≥ 4000 g), *h* Hour(s), *IDM* Infants of diabetic mothers, *IUGR* Intrauterine growth restriction, *LBW* Low birth weight (< 2500 g), *LGA* Large for gestational age, *LS* Longitudinal study, *NBW* Normal birth weight (2500–3999 g), *n.d.* No details, *NICU* Neonatal intensive care unit, *OR* Odds ratio, *SD* Standard deviation, *SGA* Small for gestational age, *VLBW* Very low birth weight (< 1500 g)

^*^Studies are sorted in descending order according to the Gross National Income (GNI) per capita of the respective countries, based on the World Bank classification [42]:

High-income countries (HIC): GNI ≥ $13,936

Upper-middle-income countries (UMIC): GNI 13,935

Lower-middle-income countries (LMIC): GNI 4,495

Low-income countries (LIC): GNI ≤ $1,135

## Risk factors and vulnerable groups

Both neonatal hypothermia and hypoglycemia arise from multifactorial origins and are closely linked to the physiological challenges encountered during the transition from intrauterine to extrauterine life. A substantial overlap exists in the risk factors for both conditions, which frequently co-occur in the neonatal population. Therefore, identifying shared risk factors and vulnerable groups is critical for implementing preventive strategies and targeted early interventions.

Prematurity is among the most prominent shared risk factors. Preterm neonates are particularly susceptible to both hypothermia and hypoglycemia due to immature thermoregulatory mechanisms, underdeveloped skin barriers, limited subcutaneous fat, and diminished glycogen stores. Their increased surface-area-to-mass ratio further exacerbates heat loss and energy demand, thereby enhancing the risk of metabolic decompensation [1, 4, 11, 43].

A similar vulnerability is observed in SGA and IUGR infants, who also represent a particularly high-risk group because of reduced fat mass and hepatic glycogen stores, impaired thermogenesis, and altered endocrine responses [44, 45]. I.e., postnatal transient hyperinsulinism appears to play a central role in the development of hypoketotic hypoglycemia [46]. In a study performed by Lazic-Mitrovic et al., hypothermia was identified as a significant early predictor of hypoglycemia in term IUGR neonates, illustrating the heightened susceptibility of this population [9, 28].

Another risk factor is delayed or insufficient postnatal energy intake. Late initiation of feeding or early feeding difficulties impair glucose homeostasis, as both thermogenesis and basal metabolism are heavily reliant on adequate energy supply [11]. Likewise, the absence of skin-to-skin contact in the immediate postnatal period deprives the neonate of exogenous warmth and emotional stabilization, thereby increasing metabolic expenditure and reducing glucose reserves [11, 47, 48].

Furthermore, perinatal stress—resulting from conditions such as birth asphyxia, cesarean section without labor, maternal diabetes, low Apgar scores, or early-onset sepsis—can significantly impair thermoregulatory and metabolic adaptation. These factors are associated with altered hormonal stress responses, including insufficient counter regulation, which compromises both thermogenesis and glycemic stability [1, 4, 11, 49, 50]. Exposed neonates frequently develop temperature instability and disrupted energy utilization and therefore require close monitoring for evolving hypothermia and hypoglycemia.

Infants of diabetic mothers frequently develop postnatal hypoglycemia due to fetal hyperinsulinism and abrupt withdrawal of maternal glucose supply. Their thermoregulatory responses may also be compromised due to delayed metabolic adaptation [21, 45].

Neonates born in low-resource settings, particularly those delivered at home or in facilities without adequate thermal care infrastructure, are also at significantly increased risk. Several studies from Ethiopia and Nepal included in this review reported rates of neonatal hypothermia up to 81%, often accompanied by high incidences of hypoglycemia and related complications [17, 20, 22, 29, 38, 40, 41].

In conclusion, identification of risk factors and vulnerable populations is essential to mitigate the burden of hypothermia and hypoglycemia in neonates by incorporating this knowledge in management guidelines. Targeted preventive interventions such as early skin-to-skin care, timely initiation of feeding, structured monitoring protocols, and environmental thermal control must be prioritized, especially for high-risk groups. Improved recognition and understanding of these overlapping vulnerabilities may enhance early neonatal outcomes and reduce the incidence of preventable metabolic complications.

## Thermal care strategies and their impact on neonatal glycemic stability

Due to its high prevalence and significant health impact, neonatal hypothermia has become a growing focus of neonatal research in recent years. In this context, numerous studies have investigated the effects of various thermoregulatory interventions on a broad range of neonatal outcomes. Although the relationship between hypothermia and hypoglycemia is complex and multifactorial, it has prompted investigation into whether early thermal management can improve postnatal glucose stability. The following section provides an overview of the current evidence on the role of early thermal care in supporting glycemic stability. This section emphasizes the importance of the “golden hour”—the first 60 min after birth—which is critical for achieving effective thermal and metabolic stabilization. It also discusses the impact of specific interventions and the relevance of structured clinical protocols and guidelines.

### The significance of the golden hour

The interplay between hypothermia and hypoglycemia is particularly relevant during the immediate postnatal period, when neonates experience a sudden drop in ambient temperature and an abrupt cessation of transplacental glucose supply following cord clamping. These simultaneous stressors can rapidly destabilize thermal and metabolic homeostasis. Timely and appropriate management in the first minutes of life is therefore essential to allow effective thermal and metabolic adaption [51]. In this context, the concept of the “golden hour” has emerged as a central element of neonatal care. It refers to the first 60 min after birth—a critical window during which targeted, evidence-based interventions can most effectively support physiological adaptation to extrauterine life. “Golden hour” protocols emphasize coordinated measures to stabilize key functions, including temperature regulation, glucose metabolism, respiratory function, and cardiovascular status [51–53].

Although these structured care bundles were initially designed for very preterm infants, growing clinical experience suggests that late preterm and term neonates may also benefit from their systematic application [52].

Several studies have reported that implementation of “golden hour” protocols reduces the incidence of both hypothermia and hypoglycemia in extremely preterm neonates [54]. However, the observed reduction in hypoglycemia has largely been attributed to early intravenous glucose administration rather than to improved thermal regulation [51–53, 55, 56]. As such, the causal relationship between hypothermia and hypoglycemia within the context of “golden hour” care has not been explicitly investigated. Nevertheless, the underlying physiological rationale suggests that minimizing cold stress through effective thermal management could reduce glucose consumption and thereby contribute indirectly to improved glycemic stability in the early postnatal period.

### The contribution of thermoregulatory interventions on early neonatal glucose homeostasis

Thermoregulatory interventions during immediate postnatal care include early initiation of skin-to-skin contact (SSC) or kangaroo mother care (KMC), polyethylene wrapping, radiant warmers, pre-warmed blankets, and delayed bathing of the newborn. By minimizing cold stress and the need for non-shivering thermogenesis, these strategies help to preserve energy stores and limit glucose consumption, potentially impacting the incidence of neonatal hypoglycemia.

Several clinical studies have investigated the effects of early SSC or KMC on the occurrence of hypothermia and hypoglycemia. Chiruvolu et al. demonstrated that implementing SSC in late preterm and term infants at risk for hypoglycemia led to a significant reduction in NICU admissions due to low blood glucose levels [47]. Although the direct relationship between hypothermia and hypoglycemia was not analyzed in detail, the authors postulated that SSC likely supports thermoregulation and reduces the utilization of brown adipose tissue, which may contribute to more stable glucose levels. These findings are supported by a Cochrane review, which showed that healthy term infants receiving early SSC had significantly higher blood glucose levels compared to controls [48].

In a retrospective study by Chen et al. a shorter duration of SSC was associated with an increased risk of hypoglycemia in late preterm and term infants with known risk factors (e.g., SGA, LGA) [21]. Notably, the hypoglycemic group also presented with significantly lower admission temperatures than their euglycemic counterparts, suggesting a possible link between temperature instability and impaired glucose regulation). Similar results were reported by Luong et al. and Ramaswamy et al., who observed reductions in both hypothermia and hypoglycemia with the use of SSC across different neonatal risk groups (e.g., 2% vs. 70% for hypothermia and 4% vs. 24% for hypoglycemia at 180 min of life) [2, 57]. A meta-analysis by Boundy et al. further confirmed that KMC significantly reduces the incidence of both conditions, with a 78% risk reduction for hypothermia (relative risk (RR) = 0.22; 95% CI: 0.12–0.41) and a substantial reduction for hypoglycemia (RR = 0.12; 95% CI: 0.05–0.32) [58].

Another relevant postnatal intervention is the timing of the first newborn bath. Although early bathing remains standard practice in some countries, it has become increasingly controversial. A retrospective cohort study by Warren et al. found that delaying the first bath for more than 24 h after birth was associated with significantly lower rates of both hypothermia and hypoglycemia. In contrast, early bathing (< 24 h) may lead to heat loss during a vulnerable phase of thermoregulation, thereby increasing metabolic demands and the risk of hypoglycemia [1, 59, 60].

For very low birth weight infants, the use of polyethylene wraps is an effective strategy to prevent postnatal heat loss. Although this intervention is primarily used in extremely preterm populations, it provides insight into the physiological relationship between thermoregulation and glucose stability. Studies have shown that polyethylene wrapping significantly reduces both hypothermia and hypoglycemia during NICU admission, likely by preserving body temperature and reducing the metabolic demand for thermogenesis [43, 61, 62].

Finally, the systematic review by Ruan et al. identified delayed breastfeeding not only as a risk factor for neonatal hypoglycemia due to delayed energy intake, but also for hypothermia [11]. This underscores the close physiological link between thermal stability and metabolic adaptation in the early neonatal period.

In summary, although the mechanisms underlying these observations are complex and multifactorial, current evidence supports that targeted thermal protection strategies may not only reduce the risk of hypothermia but also improve glucose homeostasis and reduce the incidence of hypoglycemia during the critical early hours after birth. Structured protocols and guidelines play a pivotal role to translate these physiological insights into widespread clinical practice. The following section examines such protocols/guidelines and their incorporation of the hypothermia - hypoglycemia relationship.

### Structured protocols and guidelines

Structured clinical protocols have proven instrumental in standardizing thermal care and reducing variability in practice. Programs such as the WHO warm chain and evidence-based delivery room bundles translate individual thermoregulatory measures into coordinated, protocol-driven care [3]. These approaches support adherence to best practices, promote interprofessional consistency, and serve as a basis for systematic quality improvement initiatives.

Evidence from various clinical settings demonstrates that the implementation of structured clinical protocols can substantially reduce the incidence of neonatal hypothermia, particularly in preterm infants [52, 53, 56, 63]. For example, Harer et al. reported a reduction in hypothermia rates from 63% before to 30% after implementation, and Russo et al. observed a decrease from 55% to 6% after introducing targeted thermal management measures [54, 64, 65]. Moreover, several observational studies and quality improvement projects suggest that improved thermal care—e.g., through early SSC, delayed bathing, and regular temperature monitoring—may also be associated with a lower incidence of neonatal hypoglycemia [2, 21, 47, 48, 57–60]. However, this knowledge is only loosely reflected in current clinical guidelines. An analysis of 13 national neonatal hypoglycemia guidelines revealed that only 8 explicitly mention hypothermia as a relevant risk factor for hypoglycemia [13]. These findings underscore the limited awareness of the physiological and clinical interplay between thermal dysregulation and impaired glucose homeostasis.

To optimize early neonatal outcomes, future guidelines and protocols should more explicitly integrate thermoregulation into hypoglycemia prevention strategies. Raising awareness of this association among both clinicians and policymakers is a crucial step towards more comprehensive and effective neonatal care.

## Discussion

This review sought to critically examine the relationship between neonatal hypothermia and hypoglycemia in late preterm and term neonates. In addition to exploring their frequent co-occurrence, we also analyzed the underlying physiological mechanisms, shared risk factors, and potential consequences for early clinical management and guideline development. Evidence from diverse geographic and socioeconomic settings indicates that hypothermia is frequently associated with an increased risk of hypoglycemia. However, these findings must be interpreted with caution due to considerable methodological limitations and heterogeneity across the available literature.

A consistent observation, particularly from low- and middle-income countries, is the high prevalence of both conditions in early neonatal life. Rates of hypothermia ranging from 20% to over 80% and hypoglycemia rates exceeding 30% in some cohorts highlight the widespread nature of these disorders and their clinical overlap [28, 29, 34, 38, 41]. Studies such as those conducted by Wodajo et al. (2024) and Sertsu et al. (2022) provide compelling statistical evidence for a significant association between the two conditions, with hypothermic neonates showing a markedly increased odds of hypoglycemia [17, 20].

However, a major limitation of the available data is the predominance of observational study designs. Most included studies are cross-sectional, retrospective cohort studies, or case-control analyses, which are inherently limited in their ability to establish causality (see Table 1). While many reports demonstrate a significant correlation between low admission temperature and subsequent hypoglycemia, few address the temporal sequence of events in sufficient detail to determine whether hypothermia directly contributes to the development of hypoglycemia, or vice versa. Moreover, potential confounding factors, such as delayed feeding, maternal diabetes, or perinatal complications, are often not adequately controlled for.

An additional concern is the considerable heterogeneity in definitions and thresholds used for both hypothermia and hypoglycemia. Temperature thresholds range from < 36.5°C to < 35.5°C, and glucose cut-offs vary between < 2.2 mmol/L and < 2.8 mmol/L across studies (see Table 1). This inconsistency limits meaningful comparisons and generalizability of findings. Importantly, there is a lack of high-quality evidence delineating specific temperature cut-offs at which the risk of hypoglycemia begins to rise significantly. It remains unclear whether mild hypothermia alone (< 36.5°C but ≥ 36.0°C) is sufficient to impair glucose homeostasis, or whether more pronounced hypothermia is required.

Furthermore, the literature is geographically imbalanced. Most studies have been conducted in low-resource settings, where both thermal care and glucose monitoring may be suboptimal (see Table 1). While these studies offer valuable insight into the burden of disease and clinical risk factors in these environments, they may not fully reflect the conditions and care practices in high-income countries. The relatively limited number of studies from high-income settings, such as those by Nguyen et al. (Canada), Saw et al. (Australia), and Petersen et al. (Germany), underscores the need for further research in more advanced healthcare systems, where early postnatal care protocols may mitigate some of the observed risks [18, 31, 32].

Lastly, research investigating the combined effect of hypothermia and hypoglycemia on long-term neurodevelopmental outcomes is lacking. As both conditions independently increase the risk of neurological impairment, their potential synergistic impact warrants further investigation [15]. Notably, few studies have systematically assessed the effect of specific interventions on glycemic outcomes, and vice versa, in controlled experimental settings.

## Conclusion

The current evidence base on the relationship between neonatal hypothermia and hypoglycemia in late preterm and term born neonates is limited by methodological variability, inconsistent definitions, and a predominance of data from low-resource settings. Most available studies are observational in nature and do not allow robust conclusions about causality, however, they support a plausible and clinically relevant association between hypothermia and hypoglycemia. Therefore, further research, particularly prospective clinical studies comparing actual standard of care with more advanced concepts of thermal management or evaluating efficacy of structured quality improvement plans, might provide evidence-based guidance for clinical practice and improve neonatal outcomes.

## Data Availability

The datasets used and/or analyzed during the current study are available from the corresponding author on reasonable request.

## Abbreviations

AOR: Adjusted odds ratio
BG: Blood glucose
BW: Birth weight
CI: Confidence interval
CSS: Cross-sectional study
ELBW: Extremely low birth weight (< 1000 g)
GA: Gestational age
GNI: Gross national income
HBW: High birth weight (≥ 4000 g)
HIC: High-income countries
IDM: Infants of diabetic mothers
IUGR: Intrauterine growth restriction
KMC: Kangaroo mother care
LBW: Low birth weight (< 2500 g)
LGA: Large for gestational age
LIC: Low-income countries
LMIC: Lower-middle-income countries
LS: Longitudinal study
NBW: Normal birth weight (2500–3999 g)
n.d.: No details
NICU: Neonatal intensive care unit
OR: Odds ratio
RR: Relative risk
SD: Standard deviation
SGA: Small for gestational age
SSC: Skin-to-skin contact
UMIC: Upper-middle-income countries
VLBW: Very low birth weight (< 1500 g)
WHO: World health organization
